# Chinese Medicines Induce Cell Death: The Molecular and Cellular Mechanisms for Cancer Therapy

**DOI:** 10.1155/2014/530342

**Published:** 2014-10-14

**Authors:** Xuanbin Wang, Yibin Feng, Ning Wang, Fan Cheung, Hor Yue Tan, Sen Zhong, Charlie Li, Seiichi Kobayashi

**Affiliations:** ^1^School of Chinese Medicine, The University of Hong Kong, 10 Sassoon Road, Pokfulam, Hong Kong; ^2^Laboratory of Chinese Herbal Pharmacology, Hubei Key Laboratory of Wudang Local Chinese Medicine Research, School of Pharmacy, Hubei University of Medicine, Shiyan, Hubei 442000, China; ^3^California Department of Public Health, 850 Marina Bay Parkway, G365, Richmond, CA 94804, USA; ^4^Faculty of Healthy Science, Hokkaido University, Kita 15, Nishi 7 Kita-ku, Sapporo, Japan

## Abstract

Chinese medicines have long history in treating cancer. With the growing scientific evidence of biomedical researches and clinical trials in cancer therapy, they are increasingly accepted as a complementary and alternative treatment. One of the mechanisms is to induce cancer cell death. *Aim*. To comprehensively review the publications concerning cancer cell death induced by Chinese medicines in recent years and provide insights on anticancer drug discovery from Chinese medicines. *Materials and Methods*. Chinese medicines (including Chinese medicinal herbs, animal parts, and minerals) were used in the study. The key words including “cancer”, “cell death”, “apoptosis”, “autophagy,” “necrosis,” and “Chinese medicine” were used in retrieval of related information from PubMed and other databases. *Results*. The cell death induced by Chinese medicines is described as apoptotic, autophagic, or necrotic cell death and other types with an emphasis on their mechanisms of anticancer action. The relationship among different types of cell death induced by Chinese medicines is critically reviewed and discussed. *Conclusions*. This review summarizes that CMs treatment could induce multiple pathways leading to cancer cell death, in which apoptosis is the dominant type. To apply these preclinical researches to clinic application will be a key issue in the future.

## 1. Introduction

Cancer is one of the leading causes of death in the world. GLOBOCAN data revealed that approximately 12.7 million new cases of cancers have been diagnosed and 7.6 million deaths were attributed to cancers in 2008 [1]. In these life-threatening cancers, the causes are diverse and complex and are only partially understood; the reasons why they are difficult to cure might be due to the complicated cancer hallmarks: sustaining proliferative signaling, resisting cell death, inducing angiogenesis, enabling replicative immortality, activating invasion and metastasis, evading growth suppressors, irregulating cellular energetic, genome instability, and mutation as well as tumor-promoting inflammation, and avoiding immune destruction, among which resisting cell death is the intracellular or external factors-triggered tumor action to escape from insult [2].

Cell death has conventionally been divided into three types: apoptosis (Type I), autophagy (Type II), and necrosis (Type III) [3, 4]. Apoptosis, Type I programmed cell death (PCD), is a normal response of a physiological process; it becomes a pathological trait in many diseases including cancers when apoptosis is irregulated. It is also the major type of cell death induced by most of the frontline chemotherapeutic agents [3, 5, 6]. In the process of apoptotic cell death, cells have altered morphology such as blebbing, cell shrinkage, nuclear fragmentation, and chromatin condensation. Morphological features of Type II cell death are different from those of apoptosis, in which formation of autophagosome and cytolysis of autophagosome-lysosome fusion involve the degradation of the components in cancer cells through the lysosomal machinery [7]. Type III cell death is a necrotic process whose typical characteristics of necrosis include disruption of plasma membrane and induction of inflammation that have been conventionally regarded as an accidental, uncontrolled cell death. However, recent studies found that necrosis could be under control as it shared the same stimuli (cytokines, pathogens, ischemia, heat, and irradiation), signaling pathways (death receptors, kinase cascades, and mitochondrial), and protective mechanisms (Bcl-2/Bcl-x, heat shock protein) as apoptosis [5, 8]. Besides these three types of cell death, several other cell death pathways have been elucidated [4, 9–12]. Since these distinct cell deaths have different subroutines, the Nomenclature Committee on Cell Death (NCCD) has proposed a set of recommendations to define cell deaths based on the biochemical and functional condensation in 2012 [9].

Since many of the clinical anticancer drugs are originally from natural sources, such as vinca alkaloids and taxanes, up to date, some studies have focused on the herbal medicinal products, especially Chinese medicines (CMs, including plants, animals, and minerals) [13–18]. Natural products are important sources of anticancer lead molecules. Many successful anticancer drugs come from natural products. More are still under clinical trials. The aim is to develop novel anticancer drugs derived from natural products, especially from CMs. More critical systematic studies on cellular and molecular therapeutic principle of anticancer natural products from CMs in cancer cell deaths need to be conducted.

In this review, we retrieved the relevant publications from PubMed and other databases to summarize the actions of CMs involved in inducing cancer cell death* in vitro* and* in vivo*. Besides clinical applications, other novel cell death pathways and the relevance of CMs in these fields are also discussed here.

## 2. CMs Induce Cancer Cell Death and Their Underlying Mechanisms

### 2.1. CMs Induce Apoptotic Death in Human Cancer Cells

**Figure 1:**
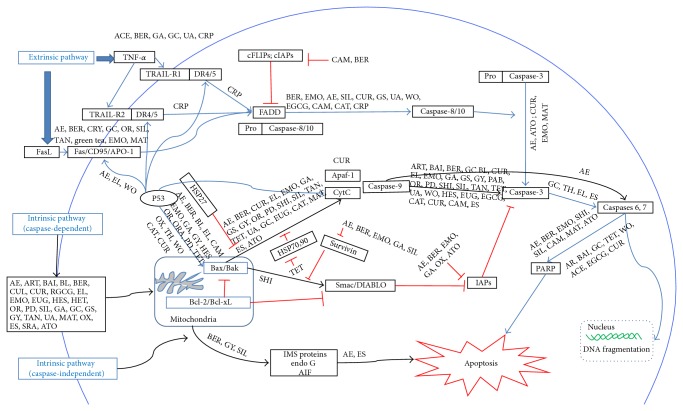
Schematic diagram of the mechanisms of CMs-induced cancer apoptosis. ACE: 1′S-1′-acetoxyeugenol acetate; AE: aloe emodin; ART: artemisinins; ATO: arsenic trioxide; BAI: baicalin; BL: baicalein; BER: berberine; CAM: camptothecin; CAT: catechins; CRP: cryptocaryone; CRY: cryptotanshinone; CUR: curcumin; CUL: curcumol; EL: *β*-elemene; EGCG: (-)epicatechin-3-gallate and polyphenols; EMO: Emodin; ES: extract of shizhuyu; EUG: eugenol; GA: gambogic acid; GC: gancao; GS: Ginseng; GY: gypenosides, HES: hesperidin; HET: hesperetin; MAT: matrine; OR: oridonin; ORA: oroxylin A; OX: oxymatrine; PD: polyphyllin D; PAB: pseudolaric acid B; SHI: shikonin; SIL: silibinin; SRA: selenium-rich amino acids; TAN: tanshinone IIA; TET: tetrandrine; TH: total huangqin glucosides; TRI: triptolide; UA: ursolic acid; WO: wogonin.

Both intrinsic and extrinsic pathways involve activation of apoptosis by CMs in human cancer cells. The CM-initiated apoptotic cell death is mainly dependent on the activation of caspase cascade. There are two types of apoptotic caspases: initiator (apical) caspases and effector (executioner) caspases. Initiator caspases (e.g., CASP2, CASP8, CASP9, and CASP10) cleave inactive proforms of effector caspases, thereby activating them. Initially, caspases are cysteine-aspartic proteases or cysteine-dependent aspartate-directed proteases in inactive forms. They are cleaved by interacting special molecules such as Apaf-1 (apoptotic protease-activating factor-1), Fas/CD95, or tumor necrosis factor *α* (TNF*α*) when apoptosis is induced in cells [9, 132]. Extrinsic apoptosis depends on caspase activation, while intrinsic apoptosis is either in caspase-dependent or -independent manner [9, 133]. CMs can activate cancer cell death extrinsically, intrinsically, or both; therefore the mechanisms of CMs inducing cancer apoptotic cell death have been more diversified.  Table 1 summarizes the general information of CMs inducing apoptotic cell death. The typical examples are in Table 1 and Figure 1.

#### 2.1.1. CMs Induce Apoptosis Intrinsically

CMs-induced intrinsic apoptosis mainly requires the activation of caspases. CMs can also induce apoptotic cell death by caspase-independent manner because some types of cancer cells can ablate the expression of caspases. In addition, even in caspase-proficient cancer cells, CMs treatment can activate all types of intrinsic apoptosis, eventually leading to potent cancer cell death.

Ursolic acid (UA) is an active ingredient in several CMs, such as* Oldenlandia diffusa* (Willd.) Roxb. (Chinese name:* Baihuasheshecao*),* Ligustrum lucidum* W.T.Aiton (Chinese name:* Nuzhen*), and* Eriobotrya japonica* (Thunb.) Lindl. (Chinese name:* Pipa*). Previous studies showed that UA could induce cancer cell death by enabling the caspase-dependent pathway. It was reported that UA activated caspase-3 and caspase-9 in human prostate cancer cells, RC-58T/h/SA#4 [32]. UA binding with oleanolic acid could elevate the caspase-3 activity in human liver cancer cells, Huh7, HepG2, Hep3B, and HA22T [35]. Its antitumor effect was also observed in xenograft model. The results of positron-emission tomography-computed tomography (PET-CT) imaging indicated that proliferation of tumor cells declined after UA treatment* in vivo* [34, 134]. Generally, the mechanism of CMs to cause intrinsic cell death in cancer is caspase-dependent. CMs induced the release of cytochrome* c* from mitochondria [23], which facilitated the activation of apoptotic protease-activating factor-1 (Apaf-1) and forms Apaf-1 apoptosome that bound to caspase-9 through CARD-CARD (caspase recruitment domain) interactions to form a holoenzyme complex [135, 136]. The complex cleaved caspase-3 to produce a caspase cascade resulting in cell death [94, 136]. The mechanisms of some representative CMs inducing cancer intrinsic cell death are illustrated in Figure 1.

Apart from caspase-dependent cell death, CMs could initiate apoptosis in both caspase-dependent and caspase-independent manners. The main biochemical pathway of caspase-independent cell apoptosis was elucidated as the results of release of mitochondrial intermembrane space (IMS) proteins and inhibition of respiratory chain. In this context, apoptosis-inducing factor (AIF) and endonuclease G (Endo G) relocated to the nucleus and mediate large-scale DNA fragmentation. The serine protease, a high temperature requirement protein A2 (HTRA2), cleaved many cellular substrates including cytoskeletal proteins as well [9]. Gypenosides (Gyp), derived from* Gynostemma pentaphyllum* (Thunb.) Makino (Chinese name:* Jiaogulan*), could suppress the growth of WEHI-3 cells* in vitro* and* in vivo* through caspase-dependent and -independent apoptosis. Gyp inhibited Bcl-2, increased Bax, and induced the release of cytochrome* c* and depolarization of mitochondrial membrane potential (Δ*ψ*) and stimulated the activities of caspase-3 and caspase-8, suggesting that Gyp triggered caspase-dependent cell death. Gyp also induced the generation of ROS and stimulated the release of AIF and Endo G, resulting in caspase-independent cell death [66]. Silibinin (from Shuifeiji, silybum marinaum (L) Gaenrt) was reported to stimulate the release of HTRA2 and AIF in bladder carcinoma cell line 5637 as well as cytochrome* c* and activate caspase-3. Thus silibinin could induce bladder cell death in both caspase-dependent and -independent manners [100] (Figure 1, Table 1).

There are some relationships between CMs and intrinsic death stimuli, for example, Scutellaria, one of the most popular CM herbal remedies, used in China and several oriental countries for treatment of inflammation, bacterial, and viral infections, and it has been shown to possess anticancer activities* in vitro* and* in vivo* in mouse tumor models [137, 138]. The bioactive components of Scutellaria were confirmed to be flavonoids [138, 139]. Chrysin is a natural flavone commonly found in honey that has been shown to be an antioxidant and anticancer agent [140]. Several studies showed that Chrysin and Apigenin could potentiate the cytotoxicity of anticancer drugs by depleting cellular GSH, an important factor in antioxidant defense [141–143]. A 50–70% depletion of intracellular GSH was observed in prostate cancer PC-3 cells after 24 h of exposure to 25 *μ*M Chrysin or Apigenin [141, 144].

#### 2.1.2. CMs Induce Apoptosis Extrinsically

Since extrinsic apoptosis of cancer cells is initiated by binding of death receptors and their ligands, the death receptors may function as signaling gateway in which Fas/CD95 ligands (FasL/CD95L) and some cytokines such as TNF*α* and TNF superfamily member 10 (TNFSF10, also known as TRAIL) play great roles in inducing apoptosis. These lethal cytokines activate Fas-associated protein with a “death domain” (FADD) and thereby activate caspase-8/10, caspase-3, caspase-6/7 to a cascade apoptosis response. Matrine, an alkaloid purified from* Sophora flavescens* Ait. (Chinese name:* Kushen*), induces the apoptosis of gastric carcinoma cells SGC-7901. A study using MTT assay showed that matrine inhibited SGC-7901 cells proliferation in dose- and time-dependent manners. Furthermore, the levels of both Fas and FasL were found to be upregulated after matrine treatment, which resulted in apoptotic cell death by the activation of caspase-3 [116]. Other CMs involved in the induction of extrinsic apoptosis included oridonin (from* Donglingcao*,* Rabdosia rubescens* (Hemsl.) Hara) [44], polyphenols from green tea [88, 89], and glycyrrhizin (from* gancao*,* Glycyrrhiza glabra* L.) [81], as listed in Table 1.

#### 2.1.3. CMs Induce Both Intrinsic and Extrinsic Apoptosis

Some of CMs exhibit a complex nature by inducing both intrinsic and extrinsic apoptosis. Kim et al. found that UA induced the expression of Fas and cleavage of caspase-3 and caspase-8 as well as caspase-9 and decreased its Δ*ψ*. Other effects, such as Bax upregulation, Bcl-2 downregulation, and the release of cytochrome* c* to the cytosol from mitochondria, were caused by UA treatment [31] (Figure 1, Table 1).

### 2.2. CMs Induce Autophagic Cancer Cell Death

**Figure 2:**
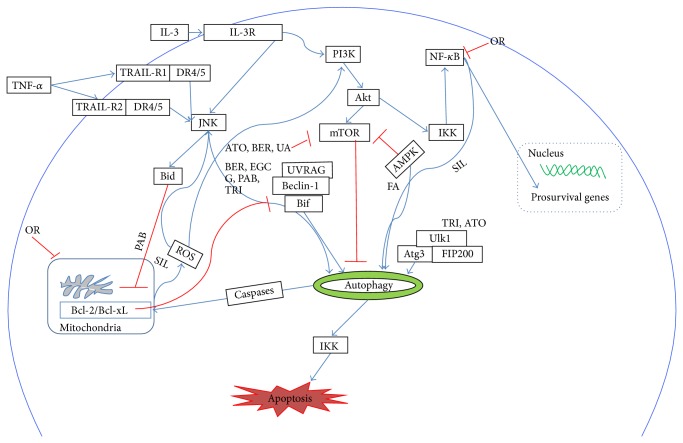
Schematic diagram of the mechanisms of the CMs for cancer autophagy death. AE: aloe emodin; ATO: arsenic trioxide; BER: berberine, EGCG: (-)epicatechin-3-gallate and polyphenols; FA: fangchinoline; OR: oridonin; PAB: pseudolaric acid BSIL: silibinin; TRI: triptolide; UA: ursolic acid.

Autophagic cell death is characterized with a massive cytoplasmic vacuolization resulting in physiological cell death, which is particularly induced when cells are deficient in essential apoptotic modulators such as Bcl-2 family and caspases. Some of the CMs induce autophagy via several signaling pathways that mediates the downregulation of mammalian target of rapamycin (mTOR) and upregulation of Beclin-1 [4, 5, 12] (Figure 2). We previously reported that fangchinoline (isolated from* Fangji*,* Stephenia tetrandra* S Moore) triggered autophagy in a dose-dependent manner on two human hepatocellular carcinoma cell lines, HepG2 and PLC/PRF/5. Blocking fangchinoline-induced autophagy process would alter the pathway of cell death leading to apoptosis; thus cell death was an irreversible process induced by fangchinoline [34]. Cheng et al. reported that the exposure of murine fibrosarcoma L929 cells to oridonin led to the release of cytochrome* c*, translocation of Bax, and generation of ROS. Additionally, oridonin induced autophagy in L929 cells through p38 and NK-*κ*B pathways. Autophagy occurred after oridonin treatment and blocking autophagy caused apoptosis [39, 40]. These observations suggested that autophagic cell death governed the cell fate upon CMs treatment. General information of CMs inducing autophagic cell death is summarized in Table 1. Figure 2 further illustrates the mechanisms of some representative CMs inducing autophagic cell death.

### 2.3. CMs Induce Necrotic Cancer Cell Death

Necrosis is classified as nonprogrammed cell death in the absence of morphological traits of apoptosis or autophagy. This phenomenon gives rise to “uncontrolled” cell death, loss of ATP, and membrane pumps [4]. In contrast to these features, recent study showed that necrosis exhibited its regulated characteristic, in other words, necroptosis [9]. This process involved alkylating DNA damage, excitotoxins, and ligation of death receptors under some conditions, which depended on the serine/threonine kinase activity of RIP1, target of a new cytoprotective agent, necrostatins. Others that affected the execution of necroptosis were named cyclophilin D, poly (ADP-ribose) polymerase 1 (PARP-1), and AIF [145]. Several researches on CMs have focused on the study of necrosis or necroptosis. Shikonin, a component extracted from* Lithospermum erythrorhizon* Siebold & Zucc. (*Zicao*), has been found to induce necrotic cell death in MCF-7 and HEK293. Han et al. reported that cell death pathway of shikonin-treated cells was different from either apoptosis or autophagic cell death in which loss of plasma membrane integrity was one of the morphology of necrotic cell death, but loss of Δ*ψ* and elevation of ROS did not critically contribute to cell death due to the protection by necrostatin-1 [106, 107]. ROS and Ca^2+^ elevated permeability transition pore complex- (PTPC-) dependent mitochondrial permeability transition (which was also induced by RIP1), while necrostatin-1 specifically prevented the cells from necroptosis. In summary, shikonin could induce cancer cells into necroptosis.

**Figure 3:**
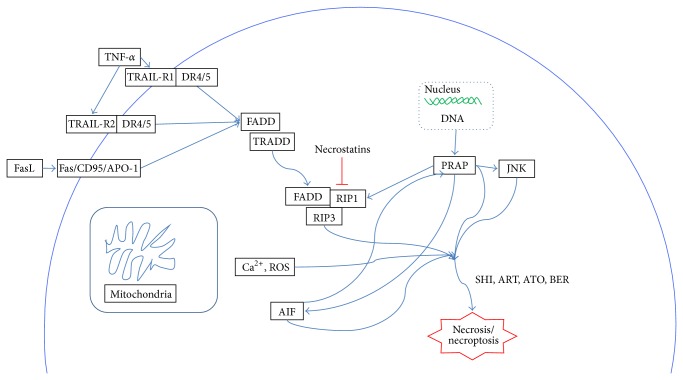
Schematic diagram of the mechanisms of CMs for cancer necrotic/necroptotic death. ART: artemisinins; ATO: arsenic trioxide; BER: berberine; SHI: shikonin.

Arsenic trioxide, another popular CM (Chinese name:* Pishuang*), also induced necrosis in the dose of 1 mg/kg accompanied by a sharp decrease of proliferation index in HCC cells [126]. Mercer et al. reported that treatment of artesunate (50 *μ*m, 48 h), an artemisinin from* Artemisia annua* L. (Chinese name:* Qinghao*), induced 24 ± 9% of necrotic/late apoptotic in HeLa cells and 67 ± 21% necrotic in HeLa *ρ* ^0^ cells. These data suggested that induced necrosis was associated with low levels of ATP and defective apoptotic mechanisms in some cancer lines [21].  Table 1 shows general information of CMs-induced necrotic cell death.  Figure 3 illustrates the mechanisms of some representative CMs-induced necrotic cell death.

## 3. Discussion

As one of the typical cancer hallmarks, cell death has attracted great attention in recent years and the study of this biological process with intervention of CMs will explore a novel way to treat cancers clinically. However, many CMs have not been approved for clinical use yet. To further investigate the efficacy and toxicity of CMs, further researches and clinical trials are necessary. In addition, a lot of CMs have been directly used as composite formula in cancer clinics according to Chinese medicine's theories for centuries. However, limited composite formula-induced anticancer action via cell death pathway is known and only few researches have been conducted from* in vitro* study, for example,* Huang-lian-jie-du-tang* (Japanese name:* oren-gedoku-to*) induced apoptotic cell death in human myeloma cells [146], HepG2, and PLC/PRF/5 cells [147]. More studies on composite Chinese medicine formula with good quality control would be needed at the molecular and cellular level.

As mentioned above, CM may exhibit integrated or additive anticancer effect through two or more subpathways. Triptolide (from* Leigongteng*,* Tripterygium wilfordii* Hook. f.) could induce both caspase-dependent and -independent apoptotic cell death by activating caspase-3, caspase-8, and caspase-9 and Bax but decreasing Bcl-2 [36–38, 113, 148–152]. These studies indicated that CMs might function on multiple modes in cancer cells which need further studies [12, 153] (Figure 1). With regard to cell deaths, through integrated or additive effect, we have conducted a study to explore how berberine (from* Huanglian*,* Coptis chinensis* Franch) induced cell death in human liver cancer cells, HepG2, and MHCC97-L. We found that the chemical induced both apoptosis and autophagy, in which autophagy accounts for 30% of berberine-induced HepG2 cell death, while apoptosis was responsible for the most contribution to liver cancer cell death. With regard to the underlying mechanism of berberine-induced autophagic and apoptotic cell death, our data demonstrated it could induce Bax activation, formation of PTPC, reduction of Δ*ψ*, and release of cytochrome* c* and Beclin-1 [111]. Similar to apoptosis, autophagy and necrosis/necroptosis affect PTPC, ROS, Ca^2+^, Bcl-2, Bax, AIF, PARP, and other cytokines during programmed cell death; it was reported that berberine induced necrosis in B16 cells [112]. But it is unknown whether berberine can induce programmed necrosis in HepG2. The cross talk among the three cell death pathways may lead to therapeutic implications. For instance, the selective inhibition of necrosis or apoptotic cell death may defend inflammation and thereby reduce subsequent tissue damage. Besides, it may serve as a novel therapeutic strategy by inducing necrotic cell death on apoptosis resistant cancer cells [109, 145].

The effectiveness of cancer chemotherapy significantly depends on apoptosis in cancer cells, while the significance of autophagy and necrosis in cancer therapy needs to be further clarified. Several reports showed that some CMs induced autophagy and inhibited cell apoptosis [30, 37, 45–48]. In contrast, some may induce autophagy leading to apoptosis [36, 41, 111]. In this context, autophagy might act as a housekeeper which eliminated abnormal proteins and recycles materials during cell starvation [7, 154]. Cell death pathway could switch to apoptosis or necrosis by inhibiting autophagy [4, 9]. However, the molecular mechanism between apoptosis and programmed necrosis (or necroptosis) is still unclear.

In addition to the above three types of cell death, there are other new types of cell death. Ginsenoside Rh2 (From* Renshen*) exhibited significant effects on cell death in colorectal cancer cells, HCT116 and SW480. Besides inducing apoptosis through activation of p53 pathway, Ginsenoside Rh2 also increased visible cytoplasmic vacuolization in HCT116 cells, which were blocked by cycloheximide (CHX), a protein synthesis inhibitor. Due to the characteristic of paraptosis as visible cytoplasmic vacuolization without disruption of the cell membrane [155, 156], Ginsenoside Rh2 was proposed as a paraptosis-like cell death inducer [42, 58, 59]. Berberine and a modified Chinese formula,* Yi Guan Jian,* might induce cancer cell anoikis [113, 149, 157]. Pharicin A (from* Xiangchacai*,* Isodon amethystoides* (Benth.) H. Hara) [123] and casticin (from* Manjing*,* Vitex rotundifolia* L.f.) [124] initiated mitotic catastrophe in cancer. Apart from the above-mentioned cell death, several other cell death pathways such as cornification, entosis, netosis, parthanatos, and pyroptosis have also been discussed elsewhere [4, 9–12]. However, to the best of our knowledge, none of the CMs is found to be involved in these novel pathways.

**Figure 4:**
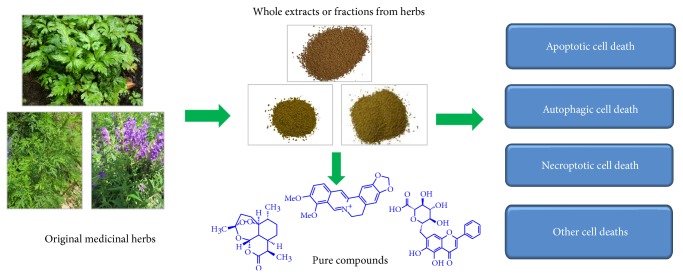
The overview of the flow chart for this review paper. The paper reviewed 45 pure compounds and extracts from CMs which can induce different cancer cell death.

In summary, this paper reviewed 45 pure compounds and extracts from CMs which can induce different cancer cell death and the underlying mechanisms. The overview of the flow chart is shown in Figure 4. Apparently, cell death is not only one mechanism of all these pure compounds and extracts for cancer therapy, but also via other mechanisms such as antiproliferation, anti-invasion, anti-angiogenesis, and anti-inflammation [15]. Since the natural sources of CMs are raw or processed materials focusing on low- or nontoxic dosages, while all these CMs in this review are pure single compounds or extracts which induce cell death by cytotoxic dosage, we should pay attention to careful explanation of the results of all these CMs. Basically, CM practitioners do not use pure compounds to treat diseases, but CM practitioners begin to integrate traditional use with results derived from modern research including characteristics of CMs inducing cell death for cancer therapy in recent years. For example, berberine, a main active compound of huanglian, is not directly used in CM clinical practice, but the various effects of berberine in cancer cell models will bring some new insight into clinical usage of huanglian when CM practitioners use huanglian combined with other herbs to treat cancer Tang et al., [158]. Usually, huanglian was used in low dosage 2–5 g to treat diseases, while high dosage of huanglian at 15–30 g was also suggested for use in recent years because we found that berberine could inhibit cancer cell migration in low dosage, while berberine could induce cell death in high dosage with safety Tang et al., [15, 111, 158]. For the high dosage of huanglian, it needs further validation by clinical study. On the other hand, limited composite formula-induced anticancer action via cell death pathway is known and only few researches have been conducted from* in vitro* study; more studies on composite Chinese medicine formula with good quality control would be needed at the molecular and cellular level and clinical studies.

## 4. Conclusions

This review showed that CMs treatment could induce multiple cancer cell death pathways including apoptosis, autophagy, necrosis, and other kinds of cell death, in which apoptosis is the most dominant type. How to apply these preclinical researches to clinical application will be a key issue in the future. The summary about CMs inducing cell death in this systematic review may offer insight into future development of cancer drug discovery from CMs and clinical application of CMs in cancer treatment.

## Figures and Tables

**Table 1:** Pure compounds and fractions of CMs inducing cancer cell death and the pathways.

Compounds	Resource/Chinese name	Cell death pathway
Artemisinins	*Artemisia annua *L./*qinghao *	Apoptosis, necrosis [19–21].

Tanshinone IIA; cryptotanshinone	*Salvia miltiorrhiza* Bunge*/Danshen *	Tanshinone IIA: apoptosis [22, 23]; autophagy [24]; cryptotanshinone: apoptosis [25]

Pseudolaric acid B	*Pseudolarix kaempferi *Gordon/*Jinqiansong *	Autophagy [26]; apoptosis [27, 28]

Ursolic acid	*Oldenlandia diffusa* (Willd.) Roxb./*Baihuasheshecao;* Ligustrum lucidum W.T.Aiton/*N*ϋ*zhen*; *Eriobotrya japonica* (Thunb.) Lindl./*Pipa *	Autophagy [29, 30]; apoptosis [31–35]

Triptolide	*Tripterygium wilfordii* Hook. f./L*eigongteng *	Both apoptosis and autophagy [36]; autophagy [37]; apoptosis [38]

Oridonin	*Rabdosia rubescens* (Hemsl.) Hara/*Donglingcao *	Autophagy [39, 40]; both autophagy and apoptosis [39, 41, 42]; apoptosis [43, 44]

*β*-Elemene; curcumol	*Curcuma wenyujin* Y.H.Chen and C.Ling/*Ezhu *	*β*-Elemene: apoptosis [45–49] Curcumol: apoptosis [50]

Rp1, Rg3, Rh2, Rk1, Rg5, etc.	*Panax ginseng* C.A.Mey./*Renshen *	Extracts: apoptosis [51–55]; Rg3: apoptosis (via decrease of Pim-3 and pBad; NF-*κ*B inactivation)[56, 57]; Rh2: apoptosis and paraptosis-like cell death [42, 58, 59]; apoptosis [60]; Rp1: paraptosis [61]; apoptosis [62]; KG-135 with etoposide (formula of Rk1, Rg3 and Rg5): apoptosis [63]

Polyphyllin D	*Paris polyphylla* Sm./*Chong Lou *	Apoptosis [64, 65]

Gypenosides	*Gynostemma pentaphyllum* (Thunb.) Makino/*Jiaogulan *	Apoptosis [66]

Baicalin; wogonin; oroxylin A; baicalein	*Scutellaria baicalensis* Georgi./*Huangqin *	Apoptosis [67–75]

Hesperidin	*Citrus reticulate* Blanco./*Chenpi *	Apoptosis [76–78]

Glycyrrhizin; 18*β*-glycyrrhetinic acid	*Glycyrrhiza glabra* L./*Gancao *	Apoptosis [79–81]

Eugenol	*Areca catechu* L.*/Binlang *	Apoptosis [82]

1′S-1′-acetoxyeugenol acetate	*Alpinia conchigera* Griff./*Jiebianshanjiang *	Apoptosis (via NF-*κ*B inactivation)[83]

Catechins (-(epicatechin-3-gallate (EGCG)), polyphenols	*Camellia sinensis* (L.) Kuntze*/Cha *	EGCG: autophagy [42, 58, 59, 84]; apoptosis [74, 75]; anoikis [85]; parthanatos [86]; catechin: apoptosis [87]; polyphenols (GrTP): apoptosis [88–90]

Cryptocaryone	*Cryptocarya concinna* Hance/Tunan	Apoptosis [91]

Curcumin	*Curcuma longa *L.*/Jianghuang *	Apoptosis [92, 93]

Emodin	*Rheum palmatum* L./*Dahuang *	Apoptosis [45–48, 94].

Aloe emodin	*Rheum palmatum* L./Dahuang; *Polygonum cuspidatum* Siebold & Zucc./*Huzhang *	Apoptosis [95, 96]

Silibinin	*Silybum marianum* (L.) Gaertn./*Shuifeiji *	Apoptosis [97–100]; autophagy [46, 101]

Gambogic acid	*Garcinia hamburgy* Hook. f./*Tenghuang *	Apoptosis [102–104]

Shikonin	*Lithospermum erythrorhizon* Siebold & Zucc./*Zicao *	Apoptosis [105]; necroptosis [106, 107]

Berberine	*Coptischinensis* Franch/*Huanglian *	Apoptosis [108, 109]; autophagy [110, 111]; necrosis [112]; anoikis [113]

Camptothecin	*Camptotheca acuminate* Decne./*Xishu *	Apoptosis [114]

Tetrandrine; fangchinoline	*Stephania tetrandra* S. Moore*/Fangji *	Tetrandrine: apoptosis [50, 115]; fangchinoline: autophagy [34]

Matrine; oxymatrine	*Sophora flavescens* Ait./*Kushen *	Matrine: apoptosis [116, 117]; autophagy [118–120]; oxymatrine: apoptosis [121]

Herbal extracts	*Zanthoxylum ailanthoides *Siebold & Zucc./*Shizhuyu *	Apoptosis [122]

Pharicin A	*Isodon amethystoides* (Benth.) H. Hara,/*Xiangchacai *	Mitotic catastrophe [123]

Casticin	*Vitex rotundifolia* L.f./*Manjing *	Mitotic catastrophe and apoptosis [124]

Selenium-rich amino acids	silkworm pupas/*Chanyong *	Apoptosis [125]

Arsenic trioxide	*Pishuang *	Necrosis [126]; apoptosis [45–48, 127–130]; autophagy [131]
